# Low phase angle in hepatobiliary and pancreatic surgery inpatients and its impact on clinical outcomes: A cross-sectional study

**DOI:** 10.1097/MD.0000000000048379

**Published:** 2026-04-17

**Authors:** Jian-Yong Cui, Jin-Dong Ma, Xiao-Rong Liu, Zhi-Wei Liu, Ji-Yue Zhang, Chun-Hui Zheng, Qiang Wei, Qiang-Pu Chen

**Affiliations:** a Department of Clinical Nutrition, Binzhou Medical University Hospital, Binzhou, Shandong, China; b Department of Hepatobiliary Surgery, Binzhou Medical University Hospital, Binzhou, Shandong, China; c Department of Nursing, Jinan Vocational College of Nursing, Jinan, Shandong, China.

**Keywords:** hepatobiliary and pancreatic surgery, inpatients, nutritional status, phase angle, sarcopenia

## Abstract

This study investigates abnormalities in bioimpedance phase angle (PA) among hepatobiliary and pancreatic surgery inpatients and analyzes their impact on clinical outcomes for surgical patients. A cross-sectional study method was employed to analyze general data, body mass index (BMI), grip strength, step speed, appendicular skeletal muscle index (ASMI), serology-related indices, body composition analysis, and NRS 2002 scores of hepatobiliary and pancreatic surgery inpatients admitted between December 2019 and September 2022. Comorbidity with sarcopenia was determined for each patient. Regarding PA, a bioelectrical impedance of <5.0° in men and <4.6° in women at 50 kHz was judged to be abnormal. The relationship between PA and sarcopenia and nutritional assessment indicators was examined with the help of univariate and multivariate logistic regression. A total of 1305 inpatients met the inclusion criteria, with 334 (25.59%) having a low phase angle. The PA abnormality rate was 6.57% for the participants under 60 years old, and 41.49% for those 60 or over. There were 349 patients with malignant disease and 956 with benign disease, and the rate of PA abnormalities in patients with malignant tumors was 28.94% and 24.37%. In 71 emaciated cases, 567 normal body masses, and 687 overweight body masses, the abnormal PA rates were 71.83%, 30.51%, and 16.01%, respectively. BMI, serum albumin level, hemoglobin level, NRS 2002 score, and age were identified as factors influencing PA (*P* < .05). Compared with the normal PA group, the low PA group had significantly decreased handgrip strength, ASMI, and skeletal muscle mass. The low PA group also exhibited a significantly higher prevalence of nutritional risk and sarcopenia compared to the control group (*P* < .001). Among the 720 surgical patients, the incidence of postoperative complications was significantly higher in the low PA group (33.60%) than in the normal PA group (17.48%) (*P* < .001), with prolonged postoperative hospital stay (*P* < .001). The prevalence of low PA was notably high among hospitalized patients who underwent hepatobiliary and pancreatic surgery. A low PA status predicts a higher nutritional risk and poorer muscle strength and function. Furthermore, low PA is associated with increased postoperative complication rates and prolonged hospitalization in surgical patients.

## 1. Introduction

Bioelectrical impedance analysis is a widely adopted method for measuring human body composition in clinical practice. It is simple, rapid, and noninvasive, making it ideal for routine use. The PA – the arctangent of the ratio of reactance to resistance measured via bioelectrical impedance analysis – serves as an important indicator of cell membrane integrity and the balance of intracellular, extracellular, and interstitial fluid.^[1,2]^ Its value represents the electrical properties of the cell membrane and provides insights into cell quality and health status. Low PA values are typically associated with malnutrition, impaired immune function, and cell membrane damage, whereas higher values reflect better health status and cellular function.^[3]^ In clinical practice, phase angle measurements have been applied to oncology, liver cirrhosis, cardiovascular disease, nephrology, chronic wasting diseases, and critical illness to assess systemic conditions and determine prognosis.^[4–9]^

Currently, research on the application of phase angle in surgical clinical practice is limited, and its clinical value and significance remain unclear. Therefore, this study analyzed preoperative PA measurements from inpatients undergoing hepatobiliary and pancreatic surgery at our center and compared them with relevant clinical and laboratory indicators. This study aimed to investigate the incidence of low PA, analyze its influencing factors, and evaluate its clinical relevance as a preoperative assessment tool.

## 2. Methods and materials

### 2.1. Research participants

This cross-sectional study included 1305 patients hospitalized in the hepatobiliary and pancreatic Surgery Department of a tertiary hospital between December 2019 and September 2023. All of these patients met the inclusion and exclusion criteria. This study was approved by the Ethics Committee of Ethics Committee of Binzhou Medical University Affiliated Hospital (Approval Number: KYLL-361). All participants were fully informed of the study purpose, procedures, potential risks, and rights, and voluntarily signed the written informed consent form prior to participation.

Inclusion criteria: patient age ≥ 18 years; patient admitted for initial treatment of hepatobiliary, or pancreatic tumors; portal hypertension; obstructive jaundice; gallstones; or chronic cholangitis/pancreatitis; no surgical intervention within the past 6 months; ability to complete sarcopenia diagnostic testing; and voluntary participation in this study.

The exclusion criteria were as follows: presence of muscle-related disorders, such as myasthenia gravis, that affect grip strength measurement; history of stroke or motor neuron disease that impairs mobility and prevents completion of the 6-Minute Walk Test; implantation of cardiac pacemakers or metal stents that interfere with bioimpedance analysis; acute onset or severe disease condition; and patients with amputations.

### 2.2. Research methods

Initially, the patients’ name, sex, age, number of hospital admissions, and diagnosis were recorded. Within 2 days of admission, trained investigators completed all preoperative surveys and measurements. After surgery, postoperative complications, hospital stay duration and hospitalization costs were also recorded.

#### 2.2.1. Height and weight measurement and body mass index calculation

Patient height and weight were measured in an upright position. The study participants were measured barefoot in the morning after fasting and after emptying their bowels and bladders. Body mass index (BMI) was calculated using the formula: BMI = weight (kg)/ height (m)^2^. Referencing BMI classification standards,^[10]^ participants were categorized into 3 groups: <18.5 (underweight), 18.5 to 23.9 (normal weight), and ≥24 (overweight).

#### 2.2.2. Body composition analysis and phase angle measurement

The PA value was determined using an InBodyS10 instrument manufactured by Biospace Co., Ltd., South Korea. Prior to measurement, participants were instructed to do the following: fast for at least 8 hours, refrain from intravenous fluids, empty their bowels and bladder, remove all electronic devices and metal jewelry, lie supine barefoot, extend their arms outward at approximately a 15° angle from the torso, position their legs shoulder-width apart, and remain flat for 10 minutes before measurement. Skeletal muscle mass, fat-free mass, and limb skeletal muscle mass were recorded. According to the Kyle^[11]^ standard, a bioelectrical impedance value of <5.0° for males and <4.6° for females at 50 kHz indicates low PA. Patients were categorized into low and normal PA value groups using this standard.

#### 2.2.3. Calculation of the appendicular skeletal muscle index

The appendicular skeletal muscle index (ASMI) was used to measure the skeletal muscle mass of the patient’s limbs using a body composition analyzer, calculated according to the ASMI formula: ASMI = appendicular skeletal muscle mass (kg)/ height^2^ (m^2^).

#### 2.2.4. Grip strength testing

Grip strength was measured using a Jamar dynamometer (Sammons Preston Inc., Bolingbrook). Each participant assumed a seated position with the elbows flexed at 90°, and they gripped the dynamometer with maximum force using their dominant hand. The average of 3 measurements served as the standard for assessing grip strength.

#### 2.2.5. Nutrition risk screening

The Nutritional Risk Screening 2002 (NRS2002) was used to assess nutritional risk. An NRS2002 score of ≥3 indicates the presence of nutritional risk.

#### 2.2.6. Diagnostic criteria

The diagnostic criteria for sarcopenia were based on the 2019 Asian Working Group for Sarcopenia expert consensus recommendations.^[12]^

#### 2.2.7. Serum marker testing

On the morning following admission, fasting venous blood was drawn to perform a complete blood count and biochemical panel including hemoglobin level (HB), red blood cell count (RBC), lymphocytes (Lym), albumin (ALB), total bilirubin level (TBIL), and other parameters.

#### 2.2.8. Diagnostic criteria for complications

The diagnosis of surgical site complications followed the relevant guidelines published by the U.S. CDC.^[13]^ The diagnosis of intra-abdominal infection is based on the diagnostic criteria established by the Surgical Infection Society.^[14]^ The diagnosis of pulmonary infection is based on the diagnostic criteria established by the Infectious Diseases Society of America and the American Thoracic Society.^[15]^ Pulmonary complications included^[16]^: pulmonary infection, atelectasis, respiratory failure, asthma, pulmonary embolism, and digestive tract fistula. Pancreatic fistula diagnosis followed the criteria established by the International Pancreatic Surgery Study Group.^[17]^ Biliary fistula diagnosis adhered to the criteria of the International Hepatic Surgery Study Group.^[18]^ The diagnosis of enterocutaneous fistula is based on the diagnostic criteria established by the American Society of Colon and Rectal Surgeons.^[19]^ Gastroparesis was diagnosed based on the criteria established by the International Pancreatic Surgery Study Group.^[20]^

#### 2.2.9. Postoperative data collection

Medical records were reviewed to collect data on postoperative complications in patients who underwent surgery. Complications were classified according to the Clavien–Dindo classification system,^[21]^ and the postoperative hospital stay duration was documented.

### 2.3. Statistical analysis

Data analysis was performed using IBM SPSS Statistics for Windows, version 26.0 (IBM Corp., Armonk). For intergroup comparisons, quantitative data underwent Kolmogorov-Smirnov normality testing. Normally distributed variables are expressed as (mean ± standard deviation) and were compared using *t*-tests. Non-normally distributed variables are presented as median (interquartile range) [M (P25–P75)] and were compared using the nonparametric Mann–Whitney *U* test. Categorical variables are expressed as frequencies (percentages), and comparisons between groups were performed using chi-square tests. Univariate and multivariate logistic regression analyses were conducted to examine the relationships between PA and nutritional assessment indicators (categorical variables), with PA as the independent variable and nutritional assessment outcomes or clinical data as the dependent variables. All tests were two-tailed, and statistical significance was set at *P* < .05.

## 3. Results

### 3.1. Low PA incidence rate

A total of 1305 participants were included in the study, comprising 682 males (52.26%) and 623 females (47.74%), with a mean age of (59.87 ± 13.57) years. Patients with low PA: 334 cases (25.59%). Among males, 170 (24.93%) had low PA, and among females, 164 (26.32%) had low PA. Non-elderly patients (<60 years old): 594 cases; elderly patients (≥60 years old): 711 cases. The PA abnormality rates were 6.57% and 41.49%. Among the 349 malignant disease patients and 956 with benign disease, the PA abnormality rates were 28.94% and 24.37%, respectively. The BMI categories included 71 underweight (71.83% PA abnormalities), 567 normal weight (30.51%), and 687 overweight (16.01%) patients. 317 patients with liver disease (19.87% PA abnormalities), 682 patients with biliary tract disease (24.63%), 306 patients with pancreatic disease (17.65%). A total of 720 surgical patients were included (17.36% PA abnormalities). Among them, 544 underwent laparoscopic surgery (13.42%), while 176 underwent open surgery (29.54%) (Table 1).

**Table 1:** Incidence of low PA in 1305 patients.

Variable	Total cases	Low PA group	Normal PA group	Low PA occurrence rate (%)
Total cases	1305	334	971	25.59
Gender				
Male	682	170	512	24.93
Female	623	164	459	26.32
Age [(years, *x̅* ± s)]	59.87 ± 13.57	70.43 ± 10.36	56.24 ± 12.61	
<60	594	39	555	6.57
≥60	711	295	416	41.49
Benign or malignant disease				
Benign disease	956	233	723	24.37
Malignant disease	349	101	248	28.94
BMI				
<18.5	71	51	20	71.83
18.5–23.9	567	173	374	30.51
≥24	687	110	577	16.01
Disease type				
Liver	317	63	254	19.87
Biliary system	682	168	514	24.63
Pancreas	306	54	252	17.65
Surgery				
Yse	720	125	595	17.36
No	585	94	491	16.07
Surgical procedure				
Laparoscopic	544	73	471	13.42
Open-abdominal	176	52	124	29.54

BMI = body mass index, PA = phase angle.

### 3.2. Analysis of indicators associated with sarcopenia in the low and normal PA groups

Results showed that individuals in the low PA group were more prone to sarcopenia, exhibiting significantly lower grip strength, ASMI, and skeletal muscle mass compared to the normal PA group (*P* < .001) (Table 2).

**Table 2:** Analysis of indicators associated with sarcopenia in the low PA group.

Variable	Low PA group	Normal PA group	χ^2^/*Z*	*P*
Sarcopenia [n (%)]				
Yes	147 (44.01)	77 (7.93)	172.886	<.001
No	187 (55.99)	894 (92.07)		
Handgrip strength (kg)	20.40 (15.00–26.40)	27.80 (21.85–35.85)	−13.115	<.001
ASMI	6.02 (5.32–6.88)	6.87 (6.29–7.68)	−12.183	<.001
Skeletal muscle mass (kg)	20.80 (18.10–24.40)	24.50 (21.10–28.80)	−11.203	<.001

ASMI = appendicular skeletal muscle index, PA = phase angle.

### 3.3. PA score correlations

**Figure 1.**
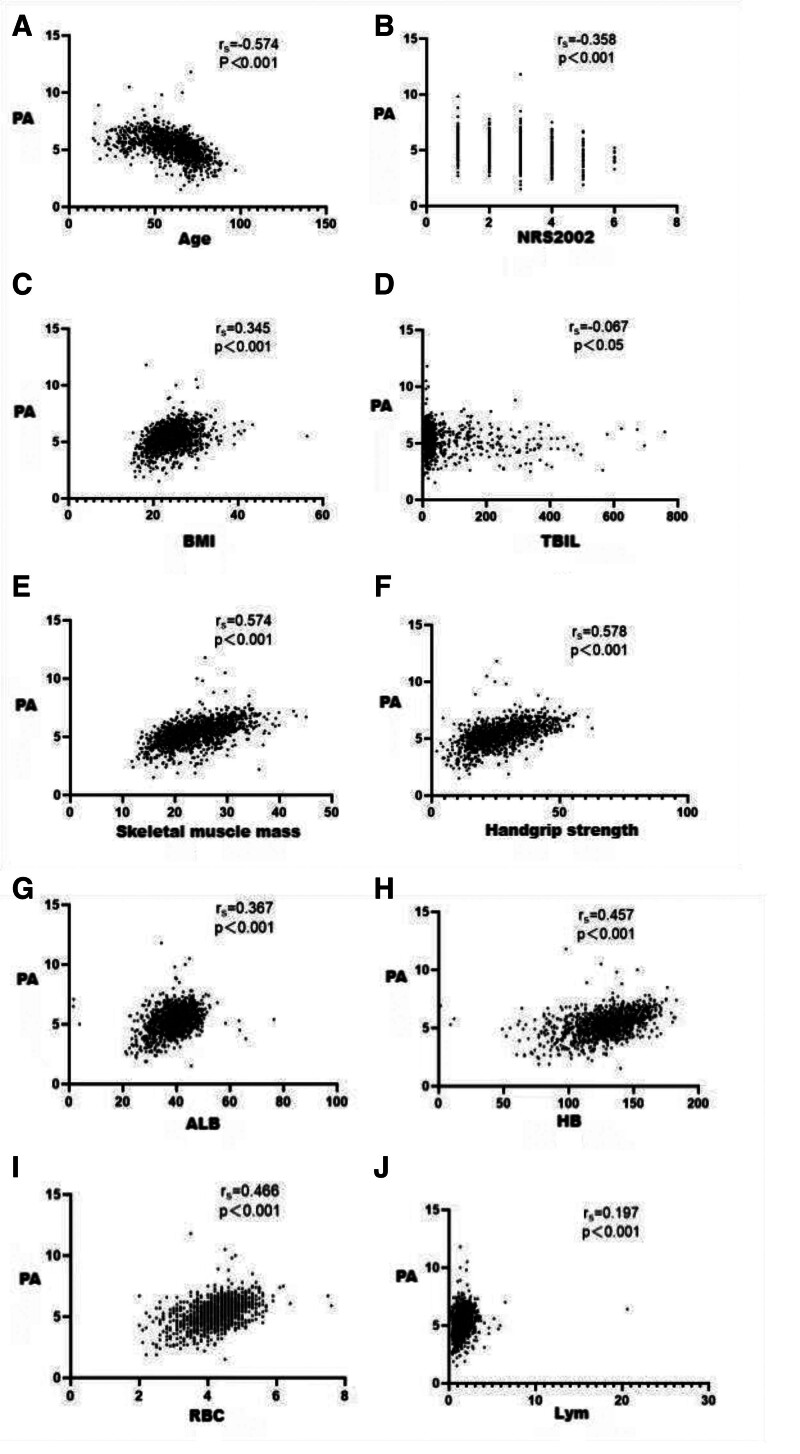
Scatter plot of PA value correlation with related variables. (A) Correlation between PA value and age; (B) Correlation between PA value and NRS2002 score; (C) Correlation between PA value and BMI; (D) Correlation between PA value and TBIL; (E) Correlation between PA value and skeletal muscle mass; (F) Correlation between PA value and grip strength; (G) Correlation between PA value and ALB; (H) Correlation between PA value and HB; (I) Correlation between PA value and RBC; (J) Correlation between PA value and Lym. ALB = albumin, BMI = body mass index, HB = hemoglobin level, Lym = lymphocytes, NRS2002 = Nutritional Risk Screening 2002, PA = phase angle, RBC = red blood cell count, TBIL = total bilirubin level.

PA values were negatively correlated with patient age, NRS2002 scores, and TBIL, and positively correlated with patient BMI, skeletal muscle mass, grip strength, ALB, HB, RBC, and Lym (*P* < .001) (Table 3 and Fig. 1).

**Table 3:** Correlations between PA and other variables.

	*r*_*s*_	*P*
Age	−0.574	<.001
NRS2002	−0.358	<.001
BMI	0.345	<.001
TBIL	−0.067	<.05
Skeletal muscle mass	0.574	<.001
Handgrip strength	0.578	<.001
ALB	0.367	<.001
HB	0.457	<.001
RBC	0.466	<.001
Lym	0.197	<.001

ALB = albumin, BMI = body mass index, HB = hemoglobin level, Lym = lymphocytes, NRS2002 = Nutritional Risk Screening 2002, RBC = red blood cell count, TBIL = total bilirubin level.

### 3.4. Analysis of factors affecting low PA

Elderly patients were more prone to phase-angle abnormalities. Patients in the low PA group exhibited significantly lower levels of ALB, HB, RBC, and Lym compared to the normal PA group (*P* < .001). Additionally, individuals in the low PA group were significantly more susceptible to nutritional risk (*P* < .001) (Table 4).

**Table 4:** Analysis of factors associated with low PA.

Variable	Low PA group (n = 334)	Normal PA group (n = 971)	χ^2^/*Z*	*P*
Age [n (%)]				
<60	39 (11.68)	555 (57.16)	207.290	<.001
≥60	295 (88.32)	416 (42.84)		
BMI [n (%)]				
<18.5	45 (13.47)	17 (1.75)	123.286	<.001
18.5–23.9	199 (59.58)	472 (48.61)		
≥24	90 (26.95)	482 (49.94)		
NRS2002 [n (%)]				
≥3	186 (55.69)	227 (23.38)	119.937	<.001
< 3	148 (44.31)	744 (76.62)		
ALB (g/L)	35.75 (31.48–39.60)	40.40 (37.20–43.80)	−12.594	<.001
HB (g/L)	119.50 (106.00–133.00)	134.00 (122.00–146.00)	−11.563	<.001
RBC (×10^12^/L)	3.90 (3.50–4.30)	4.40 (4.00–4.70)	−12.261	<.001
Lym (×10^12^/L)	1.20 (0.90–1.60)	1.50 (1.10–2.00)	−7.640	<.001
Jaundice [n (%)]				
Yes (≥17.10 μmol/L)	204 (61.08)	461 (47.48)	18.396	<.001
No (<17.10 μmol/L)	130 (38.92)	510 (52.52)		

ALB = albumin, BMI = body mass index, HB = hemoglobin level, Lym = lymphocytes, NRS2002 = Nutritional Risk Screening 2002, PA = phase angle, RBC = red blood cell count, TBIL = total bilirubin level.

### 3.5. Binary logistic regression analysis

In the binary logistic regression analysis, the dependent variable was the presence of a normal phase angle in inpatients undergoing abdominal surgery, while the independent variables were the factors that showed statistically significant differences. The results showed that BMI, ALB, and HB exerted significant positive effects on PA (a one-unit increase in BMI, ALB, or HB reduced the probability of abnormal PA values by 10.8%, 6%, and 1.3%, respectively). NRS2002 scores and age significantly negatively correlated with PA (each 1-unit increase in NRS2002 score and age increased the probability of abnormal PA by 19.9% and 10.8%, respectively). Patients with sarcopenia had a 1.679-fold higher probability of PA abnormalities compared to non-sarcopenic patients, representing a significant independent risk factor. However, Lym, RBC, and TBIL levels did not produce statistically significant differences in PA values (Table 5).

**Table 5:** Binary logistic regression analysis.

Variable	Regression coefficient	Wald χ^2^	*P*	OR	95% CI
Sarcopenic	0.518	6.986	.008	1.679	1.143–2.467
NRS2002	0.182	8.829	.003	1.199	1.064–1.352
BMI	−0.114	18.388	<.001	0.892	0.847–0.940
ALB	−0.062	16.539	<.001	0.940	0.912–0.968
Lym	0.113	0.781	.38	0.893	0.649–1.148
HB	−0.014	3.880	.049	0.987	0.973–1.000
TBIL	−0.001	0.285	.59	1.001	0.999–1.002
RBC	0.309	1.573	.21	0.734	0.453–1.190
Age	0.103	109.320	<.001	1.108	1.087–1.129

ALB = albumin, BMI = body mass index, CI = confidence interval, HB = hemoglobin level, Lym = lymphocytes, NRS2002 = Nutritional Risk Screening 2002, OR = odds ratio, PA = phase angle, RBC = red blood cell count, TBIL = total bilirubin level.

### 3.6. Impact of PA on surgical outcomes

Among the 1305 patients, 720 (55.17%) underwent surgical treatment. The results showed significant differences in the overall postoperative complication rates between the low and normal PA groups (*P* < .001). In particular, significant differences were observed in the incidence of infectious complications, wound-healing complications, gastric paralysis, and overall Clavien–Dindo complication grading (*P* < .001). Pulmonary complications, gastrointestinal fistulas, deep vein thrombosis of the lower extremities, and hospitalization costs were not significantly different between the groups (*P* = .065) (Table 6).

**Table 6:** Impact of PA on surgical outcomes (n,%).

	Low PA group (n = 125)	Normal PA group (n = 595)	χ^2^/*z*	*P*
Overall complication rate	42 (33.60)	104 (17.48)	16.607*	<.001
Infectious complications	26 (20.80)	61 (10.25)	10.819*	.001
Pulmonary complications	14 (11.20)	52 (8.74)	0.751*	.39
Incision complications	9 (7.20)	10 (1.68)	12.248*	<.001
Digestive tract fistula	2 (1.60)	8 (1.34)	0.000*	.99
Gastric paralysis	7 (5.60)	5 (0.84)	14.279*	<.001
Lower-extremity deep vein thrombosis	1 (0.80)	3 (0.50)	0.164*	.69
Clavien–Dindo				
Ⅰ	20 (16.00)	59 (9.92)	3.914*	.048
Ⅱ–Ⅴ	22 (17.6)	45 (7.56)	12.330*	<.001
Postoperative hospital stay	8.02 ± 6.128	5.94 ± 5.488	5.117†	<.001
Hospitalization expenses	2.73 ± 1.82	2.71 ± 2.03	0.519†	.60

PA = phase angle.

* χ^2^ test.

† Mann-Whitney *U*.

## 4. Discussion

This study measured bioimpedance PA values in inpatients undergoing hepatobiliary and pancreatic surgery. The incidence of low PA across the entire patient group was found to be 25.59%. Among elderly patients, the incidence of low PA was 41.49%, significantly higher than that observed in non-elderly patients. The incidence of low PA in malignant tumor patients (28.94%) was somewhat higher than that in benign disease patients (24.37%). This indicates that low PA is a common issue among hepatobiliary and pancreatic surgery patients and warrants increased attention from surgeons.

Previous studies have demonstrated that the bioimpedance phase angle reflects intracellular and extracellular water distribution as well as somatic cell mass. Normal PA values indicate cellular integrity and functional normality, whereas decreased PA values suggest compromised cell integrity, reduced cellular function, and increased apoptosis.^[22]^ When the body experiences inflammation, tumors, malnutrition, or water and electrolyte imbalances, cellular dysfunction occurs, leading to abnormal PA values. Studies have shown that factors such as gender, body mass, fat-free mass, C-reactive protein, and HB influence PA values.^[23,24]^ This study found that patients in the low PA group exhibited significantly lower BMI, ALB, and HB than those in the normal PA group, suggesting that these factors may influence PA variations in patients undergoing hepatobiliary and pancreatic surgery. Normal PA levels can be maintained by addressing these factors.

Currently, clinical methods for nutritional risk screening primarily include the NRS2002 score, Subjective Global Assessment, Mini Nutritional Assessment, Controlling Nutritional Status, and BMI. However, these methods have limitations, such as insufficient precision.^[25,26]^ The bioimpedance phase angle is an objective, precise parameter that does not rely on calculation formulas. Our findings indicated that the low PA group exhibited higher NRS2002 scores, lower BMI, lower ALB, and lower HB compared to the normal PA group. This suggests that PA can reflect the nutritional status of patients, with lower PA values indicating higher nutritional risk. Huang et al^[27]^ conducted a study analyzing 140 patients with laryngeal cancer. The results indicated that the incidence of malnutrition in the low-PA group reached 71.2%, which was significantly higher than that in the normal-PA group, and was consistent with the findings of this study.

Patients with sarcopenia experience progressive decline in skeletal muscle mass throughout the body, reduced intracellular water content, increased extracellular water, and diminished function,^[28]^ all of which is closely associated with chronic conditions such as malnutrition. This study assessed sarcopenia in hepatobiliary and pancreatic surgery inpatients using the Asian sarcopenia diagnostic criteria, measuring muscle mass via body composition analysis, grip strength, and the 6-meter walk test. Results showed that the incidence of sarcopenia in the low PA group (44.01%) was significantly higher than that in the normal PA group. Grip strength and ASMI were also significantly lower in the low PA group than in the normal PA group, indicating that low PA predicts a higher risk of sarcopenia in patients. A study by Jiang et al^[29]^ involving 146 patients aged 65 and older demonstrated that physical activity was significantly correlated with skeletal muscle mass in the limbs, grip strength, and ASMI. Meanwhile, a study by Kołodziej et al^[30]^ including 1567 individuals revealed markedly reduced PA values in patients with sarcopenia. Therefore, PA measurement can aid in the screening and diagnosis of sarcopenia.

Among the 720 patients in this cohort who underwent surgical treatment, we examined the effect of low PA on postoperative complications and length of hospital stay. Our findings indicated that low pulmonary artery pressure significantly increases the risk of postoperative complications and prolongs the duration of hospitalization. The incidence of Clavien–Dindo grade II or higher complications, infectious complications, incisional complications, and gastric paralysis was higher, while the impact on pulmonary complications, gastrointestinal fistulas, and deep vein thrombosis was minimal. Arero et al^[31]^ conducted a meta-analysis of extracardiac surgery patients and found that low PA is a significant risk factor for postoperative complications. Gulin et al^[32]^ investigated the impact of PA on postoperative outcomes following gastrointestinal tumor surgery, revealing that patients with lower preoperative phase angle values experienced higher rates of complications within one month postoperatively and longer hospital stays, determining that a PA < 5.5° may serve as an indicator for predicting an increased risk of postoperative complications. Therefore, the preoperative measurement of PA is a significant reference value for assessing patient clinical outcomes. Low PA impairs tissue cell function, reduces body resistance, and diminishes tissue repair and healing capacity, thereby increasing susceptibility to postoperative complications. In addition, patients with low PA levels exhibit higher rates of malnutrition and sarcopenia. Research has demonstrated that malnutrition and sarcopenia are significant risk factors for postoperative complications.^[33,34]^ This study has limitations: the sample size is limited and it is a single-center study, which may introduce selection bias. The generalizability of the findings requires further validation.

This study has several limitations that should be acknowledged. First, the relatively small sample size is a major constraint. As a single-center retrospective study, we were only able to recruit a maximum of 1305 patients over the study period. This sample size may limit the statistical power of our analyses and the generalizability of our findings to broader populations or different clinical settings. Therefore, the results of this study should be interpreted with caution. Future multi-center, prospective studies with larger sample sizes are warranted to confirm our preliminary findings.

In summary, the incidence of low PA is relatively high among inpatients undergoing hepatobiliary and pancreatic surgery, particularly among elderly patients and those with tumors. Low PA is closely associated with nutritional status and sarcopenia in inpatients undergoing hepatobiliary and pancreatic surgery and serves as a significant risk factor for adverse postoperative clinical outcomes. The PA measurement method is simple and rapid, offering considerable reference value for assessing the nutritional status and sarcopenia of inpatients undergoing hepatobiliary and pancreatic surgery, and for determining their clinical outcomes.

## Acknowledgments

We are grateful to all participants who voluntarily took part in this clinical study. Their contributions are essential to the completion of this research. Special thanks to Dr Chen Qiangpu for his professional advice in the writing of this article. We would like to thank Editage (www.editage.cn) for English language editing.

## Author contributions

**Conceptualization:** Jian-Yong Cui.

**Data curation:** Jian-Yong Cui.

**Formal analysis:** Jian-Yong Cui, Jin-Dong Ma, Zhi-Wei Liu.

**Funding acquisition:** Chun-Hui Zheng.

**Investigation:** Ji-Yue Zhang.

**Methodology:** Jin-Dong Ma.

**Project administration:** Xiao-Rong Liu, Qiang-Pu Chen.

**Resources:** Xiao-Rong Liu.

**Software:** Zhi-Wei Liu.

**Supervision:** Qiang-Pu Chen.

**Validation:** Ji-Yue Zhang, Qiang Wei.

**Visualization:** Qiang Wei.

**Writing – original draft:** Jian-Yong Cui, Jin-Dong Ma.

**Writing – review & editing:** Qiang-Pu Chen.
